# Numerical Evaluation of Gaussian Mixture Entropy

**DOI:** 10.3390/e28040381

**Published:** 2026-03-30

**Authors:** Basheer Joudeh, Boris Škorić

**Affiliations:** Department of Computer Science and Mathematics, Eindhoven University of Technology, 5612 AZ Eindhoven, The Netherlands

**Keywords:** Gaussian mixture, entropy, mixture distribution, differential entropy

## Abstract

We develop an approximation method for the differential entropy h(X) of a *q*-component Gaussian mixture in $R^{n}$. We provide two examples of approximations using our method denoted by ${\bar{h}}_{C,m}^{\mathrm{Taylor}}\left(X\right)$ and ${\bar{h}}_{C}^{\mathrm{Polyfit}}\left(X\right)$. We show that ${\bar{h}}_{C,m}^{\mathrm{Taylor}}\left(X\right)$ provides an easy-to-compute lower bound to h(X), while ${\bar{h}}_{C}^{\mathrm{Polyfit}}\left(X\right)$ provides an accurate and efficient approximation to h(X). ${\bar{h}}_{C}^{\mathrm{Polyfit}}\left(X\right)$ is more accurate than known bounds and is conjectured to be much more resilient than other approximations in high dimensions.

## 1. Introduction

### 1.1. Differential Entropy of Gaussian Mixtures

A Gaussian mixture is a probability density function on $R^{n}$ of the form $f\left(x\right)=\sum_{j=1}^{q}p_{j}N_{w_{j},K_{j}}\left(x\right)$, where $x\in R^{n}$, the $p_{j}>0$ are weights satisfying $\sum_{j=1}^{q}p_{j}=1$, and $N_{w,K}$ stands for a Gaussian distribution with mean w and covariance matrix *K*. In other words, *f* is a mixture of *q* Gaussian pdfs, with arbitrary weights, allowing each Gaussian to be of different shape and displacement. A mixture like this occurs, e.g., when a stochastic process, with probability mass function $p_{1},\ldots ,p_{q}$, determines which distribution holds for x. Such situations occur in many scientific areas. Furthermore, Gaussian mixtures are often employed as function approximators, by virtue of being smooth and localized. They have been used in a wide variety of studies, e.g., on diffusion models in physics and machine learning [1,2,3,4], non-adiabatic thermodynamics [5], wireless communication [6], wireless authentication [7], Byzantine attacks [8], gene expression [9,10], dark matter kinematics [11], and quasar spectra [12]. The differential entropy h(X) of a continuous random variable X∈X, with probability density function $f_{X}$, is defined as $h\left(X\right)=-\int_{X}f_{X}\left(x\right)\ln f_{X}\left(x\right)\;dx$. It represents the continuum limit of the (discrete) Shannon entropy for the probability mass function $f_{X}\left(x_{i}\right)▵x$, where the volume ▵x is sent to zero and the infinite contribution $\ln\frac{1}{▵x}$ is subtracted. The differential entropy of a random vector admits the same functional form as in the univariate case, and our results hold more generally in *n* dimensions.

In thermodynamics, the dissipation of heat associated with the irreversible erasure of information is at least $k_{b}T\ln2$ as shown by Landauer [13]. In particular, Gaussian mixtures have been used to model the state of a bit before erasure, and their differential entropy is important to further bound the energy, hence improving upon the Landauer bound [14]. In [15], it is shown how neuronal populations encode information in the presence of an external source. The mutual information between the input signal and the stationary distribution of the neuronal populations is used to quantify the information encoded by the neurons, and positivity hinges on the time scale of the input. Although when the input evolves rapidly the mutual information vanishes, it is shown that when the input signal is of sufficiently low frequency, the stationary distribution of the neural populations is a Gaussian mixture, and evaluating its entropy accurately is crucial in that setting. In machine learning, the Active Diffusion Subsampling method [16] uses so-called particles whose distribution is approximated by a Gaussian mixture. The entropy of the mixture plays a crucial role in choosing new samples.

Analytically computing or estimating the differential entropy of a Gaussian mixture is a notoriously difficult problem. Even numerical evaluation can be problematic in high dimensions. The special case of a single Gaussian is simple and yields $h\left(X\right)=\frac{1}{2}\ln\det\left(2\pi eK\right)$.

### 1.2. Related Work

Various methods have been proposed to approximate the differential entropy of a Gaussian mixture. A loose upper bound can be obtained from the fact that a Gaussian distribution maximizes entropy, given the first and second moments. This yields [17] $h\left(X\right)\leq \frac{1}{2}\ln\det\left(2\pi e\Sigma \right)$, with $\Sigma =\sum_{i=1}^{q}p_{i}\left(w_{i}w_{i}^{T}+K_{i}\right)-\left(\sum_{i=1}^{q}p_{i}w_{i}\right){\left(\sum_{j=1}^{q}p_{j}w_{j}\right)}^{T}$. In [18], a numerical approximation was given for the case $q=2,n=1,w_{2}=-w_{1}$. A sequence of upper bounds for arbitrary *q* was obtained in [17], but only for n=1. Furthermore, the results are not in closed form, and the bounds in the sequence do not get progressively tighter. Another method is to replace the density *f* inside the logarithm by a single Gaussian $\bar{f}$ which has mean and covariance equal to the mixture. This leads to an exact expression containing the relative entropy, $h\left(X\right)=\frac{1}{2}\ln\det\left(2\pi e\Sigma \right)-D(f||\bar{f})$; one can then find approximations or bounds for the relative entropy, as in [19,20]. This is done with Monte Carlo integration, which has the drawback of being computationally demanding and not giving an analytic expression.

In [21], an approximation was obtained by performing a Taylor expansion of lnf(x) in the variables $x-w_{j}$. To avoid the need for expansion powers above 2, they introduced the trick of representing wide Gaussians approximately as the sum of several narrow Gaussians, in the *f* outside the logarithm. The Taylor expansion, as well as the splitting trick, introduces inaccuracies. As shown in [21], one can obtain a basic concavity deficit upper bound $h\left(X\right)\leq \sum_{j=1}^{q}p_{j}\ln\frac{1}{p_{j}}+\sum_{j=1}^{q}p_{j}\frac{1}{2}\ln\det\left(2\pi eK_{j}\right)$. This is significantly tighter than the above-mentioned bound $\frac{1}{2}\ln\left[{\left(2\pi e\right)}^{n}\det\Sigma \right]$; it is exact in the case of a single Gaussian, and it gets arbitrarily close to the true value of h(X) when the overlap between the components of the mixture becomes negligible. A refinement was also introduced by means of merging parts of the mixture that are clustered together. However, in configurations where the mixture components have significant overlap whilst keeping the mixture non-trivial, this bound becomes inaccurate.

In [22], tighter bounds are introduced for the differential entropy of general mixture distributions. In particular, the concavity deficit bound is refined as $h\left(X\right)\leq \;(\sup_{i}\left|\right|g_{i}-{\bar{g}}_{i}{\left|\right|}_{\mathrm{TV}})\sum_{j=1}^{q}p_{j}\ln\frac{1}{p_{j}}+\sum_{j=1}^{q}p_{j}\frac{1}{2}\ln\det\left(2\pi eK_{j}\right)$, where $\left|\right|\cdot {\left|\right|}_{\mathrm{TV}}$ denotes the total variation distance, $\left\{g_{i}\right\}$ are the components of the mixture, and ${\bar{g}}_{j}=\sum_{i\neq j}\frac{p_{i}}{1-p_{j}}g_{i}$ is the mixture complement of $g_{j}$. Calculating the total variation distances between each component and its complement becomes computationally demanding when both the number of components and the dimension are large.

### 1.3. Contributions

We introduce a new method for estimating the differential entropy of a Gaussian mixture. We approximate $f\ln\frac{1}{f}$ by a polynomial poly(f). For any positive integer *k*, the power $f^{k}={\left(p_{1}N_{1}+\ldots +p_{q}N_{q}\right)}^{k}$ can be written as a multinomial sum containing a product of powers of Gaussians. Hence, the integral ∫poly(f(x))dx can be computed analytically. This yields a systematic way to approximate and/or lower bound h(X) by analytic expressions.

- We consider polynomials that minimize the square error $\int \mathrm{weight}\left(s\right){\left[s\ln\frac{1}{s}-\mathrm{poly}\left(s\right)\right]}^{2}\;ds$ on the range of *f*, with tunable function $\mathrm{weight}\left(s\right)=s^{r}$. We observe that negative *r* gives better results than positive *r*, which is to be expected since most of the volume in $R^{n}$ has f(x) close to zero. In particular, r≈−2 performs best in the mixture configurations that we have studied, yielding relative errors in h(X) of less than 1% already for the degree-3 polynomial fit.
- We consider the truncated Taylor series $-\ln\frac{f}{m}\approx \sum_{k=1}^{\cdots }\frac{1}{k}{\left(1-\frac{f}{m}\right)}^{k}$ for some constant *m*. This too gives rise to an approximation for $f\ln\frac{1}{f}$ that is polynomial in *f*. While not as accurate as the above-mentioned fit, it guarantees that each *k*-term has the same sign if $m\geq \max_{x\in R^{n}}f\left(x\right)$, and hence it gives rise to a sequence of increasingly accurate analytic lower bounds on h(X). We observe that the relative error in h(X) is still several percent even at polynomial degree 10.

In Section 2.1, we obtain a general recipe to approximate the differential entropy via a polynomial approximation as shown in Corollary 1. In Section 2.2, we apply Corollary 1 to the Taylor series of the logarithm to obtain a specific approximation for the differential entropy. Although it is not a very accurate approximation, it serves as an easy-to-compute lower bound. In Section 2.3, we apply Corollary 1 again using a polynomial approximation of f(s)=−slns, which gives an accurate approximation to the differential entropy. In Section 3, we show numerical results of our approximations for various configurations, and in Section 4, we assess our method and give pointers towards future work.

## 2. Polynomial Approximation

### 2.1. General Polynomial

Consider a random variable $X\in R^{n}$ whose probability density function (pdf) is a Gaussian mixture with weights ${\left\{p_{i}\right\}}_{i=1}^{q}$. The Gaussian pdfs have covariance matrices ${\left\{K_{i}\right\}}_{i=1}^{q}$, and they are centered on points $w_{1},\ldots ,w_{q}\in R^{n}$.

(1) $$\begin{array}{cc} \; & f_{X}\left(x\right)=\sum_{j=1}^{q}p_{j}N_{w_{j},K_{j}}\left(x\right)={\left(2\pi \right)}^{-\frac{n}{2}}\sum_{j=1}^{q}p_{j}{\left(\det K_{j}\right)}^{-1/2}e^{-\frac{1}{2}{\left(x-w_{j}\right)}^{T}K_{j}^{-1}\left(x-w_{j}\right)}. \end{array}$$

We let $g_{i}\left(x\right)\equiv N_{w_{i},K_{i}}\left(x\right)$ denote the Gaussian pdf in the following. The differential entropy is $h\left(X\right)=-E_{X}\left[\ln f_{X}\right]$, which can be written as follows:

(2) $$\begin{array}{cc} \; & h\left(X\right)=-E_{X}\left[\ln f_{X}\right]=-E_{X}\left[\ln mm^{-1}f_{X}\right]=-\ln m-E_{X}\left[\ln\frac{f_{X}}{m}\right], \end{array}$$

where *m* can be chosen to enforce that the argument of the logarithm lies in a certain interval. We would like to approximate the entropy by means of a polynomial approximation in powers of $f_{X}$:

(3) $$-f_{X}\ln\frac{f_{X}}{m}\approx \sum_{a=1}^{C}c_{a}f_{X}^{a},$$

In other words, we consider an order-*C* polynomial approximation. We set $c_{0}=0$ to avoid diverging integrals. Equation (3) is more relaxed than truncating a series of the form $\sum_{a=1}^{\infty }c_{a}f_{X}^{a}$; i.e., we allow $\left\{c_{a}\right\}$ to depend on *C*. Using Equation (3), the differential entropy h(X) reads as follows:

(4) $$h\left(X\right)\approx -\ln m+\sum_{a=1}^{C}c_{a}\int f_{X}^{a}\left(x\right)dx,$$

and by choosing $\left\{c_{a}\right\}$ appropriately and analytically evaluating the integral, we obtain an efficient approximation for h(X). We start by rewriting the integral in Equation (4), and the result is Corollary 1.

**Lemma 1.** 
*Let ${\left\{g_{i}\left(x\right)\right\}}_{i=1}^{q}$ be given in accordance with Equation (1), $\hat{t}\equiv \left(t_{1},\ldots ,t_{q}\right)$ s.t. $t_{i}\geq 0$ and $\sum_{i=1}^{q}t_{i}=a$. Furthermore, let $M^{-1}$ and μ be given by the following:*

(5) $$\begin{array}{cc} \; & M^{-1}=\sum_{j=1}^{q}t_{j}K_{j}^{-1}, \end{array}$$

(6) $$\begin{array}{cc} \; & \mu =\sum_{j=1}^{q}t_{j}MK_{j}^{-1}w_{j}, \end{array}$$

*and then we have the following:*

(7) $$\int \prod_{j=1}^{q}g_{j}^{t_{j}}\left(x\right)dx=D\left(\hat{t}\right),$$

*where $D(\hat{t})$ is given by the following:*

(8) $$\begin{array}{cc} \; & D\left(\hat{t}\right)={\left(2\pi \right)}^{-n(a-1)/2}\left(\prod_{i=1}^{q}{\left(\det K_{i}\right)}^{-t_{i}/2}\right){\left(\det M\right)}^{1/2}e^{-\frac{1}{2}\left(\sum_{l=1}^{q}t_{l}w_{l}^{T}K_{l}^{-1}w_{l}-\mu^{T}M^{-1}\mu \right)}. \end{array}$$

**Proof.**  See Appendix A. □

It follows that the integral in Equation (4) can be rewritten as shown in the following corollary.

**Corollary 1.** 
*The differential entropy h(X) of the Gaussian mixture is approximated by the following:*

(9) h¯c→,m(X)=−lnm+∑a=1Cca∑t^∈Taat^∏i=1qpitiD(t^),

*where $T_{a}=\{\hat{t}\in Z_{+}^{q}:|\hat{t}\left|=a\right\}$ and at^=at1…tq.*

**Proof.**  See Appendix B. □

### 2.2. Taylor Series Approximation

We perform a Taylor series for the logarithm only, in order to obtain the coefficients $\left\{c_{a}\right\}$ in Equation (3). We define $Z\equiv 1-m^{-1}f_{X}\left(X\right)$ and we write:

(10) $$\begin{array}{c} -f_{X}\ln\frac{f_{X}}{m}=-f_{X}\ln\left(1-Z\right)=f_{X}\sum_{k=1}^{\infty }\frac{1}{k}Z^{k}. \end{array}$$

The range of allowed *m* values is the one that keeps *Z* in the radius of convergence of the Taylor series: |Z|<1, i.e., $m\geq \max_{x\in R^{n}}f_{X}\left(x\right)/2$. For $m=\max_{x\in R^{n}}f_{X}\left(x\right)/2$ then Z∈[−1,1), while for $m=\max_{x\in R^{n}}f_{X}\left(x\right)$ then Z∈[0,1). Furthermore, values above $\max_{x\in R^{n}}f_{X}\left(x\right)$ such as $m=\sum_{i}p_{i}g_{i}\left(w_{i}\right)$ shrink the range of *Z* from below towards 1. By performing the Taylor expansion for the logarithm, we obtain a series of the form given by Equation (3) as shown in Theorem 1.

**Theorem 1.** 
*The procedure of performing the Taylor expansion of the logarithm as detailed in Equation (10) yields the following coefficients ${\left\{c_{a}^{\mathrm{Taylor}}\right\}}_{a=1}^{C}$ of the corresponding polynomial approximation in Equation (3):*

(11) caTaylor=HC−1,a=1,(−1)a+1ma−11a−1C−1a−1,otherwise.,

*and the corresponding differential entropy approximation as given by Equation (9) can be written as follows:*

(12) h¯C,mTaylor(X)=−lnm+HC−1−m∑a=1C−1Ba+1aC−1a,

*where $H_{k}$ is the k-th Harmonic number, and $\left\{B_{a}\right\}$ are given by the following:*

(13) Ba=(−1)ama∑t^∈Taat^∏i=1qpitiD(t^).

**Proof.**  See Appendix C. □

Note that one can show that h(X) is given by the infinite series:

(14) h(X)=−lnm−m∑k=1∞∑a=0kBa+1kka,

which is not the result of an infinite power series in $f_{X}^{a}$ as evident by the *a* summation which is inside the *k* summation. Taking the first C−1 terms and changing the order of summations makes the coefficients $\left\{c_{a}^{\mathrm{Taylor}}\right\}$ dependent on *C*. Note that Theorem 1 with $m=\max_{x\in R^{n}}f_{X}\left(x\right)$ provides a lower bound for h(X). For some applications, having a bound is more important than having a good approximation.

### 2.3. Polyfit

#### 2.3.1. General Polyfit

Suppose we have a function f(s) defined on I=[a,b] that we wish to approximate via a polynomial expansion. We write

(15) $$f\left(s\right)\approx \tilde{f}\left(s\right)=\sum_{i=1}^{C}d_{I,i}s^{i},$$

and we define the error resulting from our estimation as follows:

(16) $$E=\frac{1}{b-a}\int_{a}^{b}w\left(s\right){\left(f\left(s\right)-\tilde{f}\left(s\right)\right)}^{2}ds,$$

where we consider the possibility of favoring portions of I more than others by choosing the weight function w(s) appropriately, and w(s)=1 reduces *E* to the mean squared error. Our choice of *w* and I must be such that $M_{I}$ in Lemma 2 is invertible.

**Lemma 2.** 
*Let $M_{I}$, ${\vec{d}}_{I}$, and ${\vec{z}}_{I}$ be given by the following:*

(17) $$\begin{array}{cc} \; & {\left(M_{I}\right)}_{ij}\equiv \int_{a}^{b}w\left(s\right)s^{i+j}ds, \end{array}$$

(18) $$\begin{array}{cc} \; & {\left({\vec{d}}_{I}\right)}_{i}\equiv d_{I,i}, \end{array}$$

(19) $$\begin{array}{cc} \; & {\left({\vec{z}}_{I}\right)}_{i}\equiv \int_{a}^{b}w\left(s\right)f\left(s\right)s^{i}ds, \end{array}$$

*and then for invertible $M_{I}$, the coefficients $d_{I,i}$ in Equation (15) that minimize the error function E in Equation (16) are given by the following:*

(20) $${\vec{d}}_{I}=M_{I}^{-1}{\vec{z}}_{I}.$$

**Proof.**  See Appendix D. □

#### 2.3.2. Entropy Estimate Obtained from Polyfit

We now wish to use Lemma 2 to obtain an approximation for h(X). We first apply our estimator to the function f(s)=−slns in the following lemma.

**Lemma 3.** 
*Let f(s)=−slns on I=(a,b], r∈R if a>0 and r>−3 if a=0. Let $w\left(s\right)=s^{r}$, and then the estimator $\tilde{f}\left(s\right)$ in Equation (15) is given by the following:*

(21) $$-s\ln s\approx \sum_{i=1}^{C}d_{I,i}s^{i},$$

*where $d_{I,i}$ are obtained by solving Equation (20) with $M_{I}$ and ${\vec{z}}_{I}$ given by the following:*

(22) $$\begin{array}{cc} \; & {\left(M_{I}\right)}_{ij}=\frac{b^{i+j+r+1}-a^{i+j+r+1}}{i+j+r+1},\\ \; & {\left({\vec{z}}_{I}\right)}_{i}=\frac{\left[b^{i+r+2}\left(1-\left(i+r+2\right)\ln b\right)-\left(b\to a\right)\right]}{{\left(i+r+2\right)}^{2}}. \end{array}$$

**Proof.**  It follows from Equation (15), Lemma 2, and direct calculation. □

**Corollary 2.** 
*Let f(s)=−slns on I=(0,b], and $w\left(s\right)=s^{r}$ with r>−3; then, $\left\{d_{I,i}\right\}$ are given by the following:*

(23) $$d_{I,i}=\left\{\begin{array}{cc} {\tilde{d}}_{1}-\ln b, & \mathrm{if}\;i=1\\ b^{1-i}{\tilde{d}}_{i}, & \mathrm{otherwise} \end{array}\right.,$$

*where $\vec{\tilde{d}}$ is obtained by solving*

(24) $$\vec{\tilde{d}}={\tilde{M}}^{-1}\vec{\tilde{z}},$$

*with $\tilde{M}$ and $\vec{\tilde{z}}$ given by the following:*

(25) $${\tilde{M}}_{ij}=\frac{1}{i+j+r+1},\;\;\;\;\;{\tilde{z}}_{i}=\frac{1}{{\left(i+r+2\right)}^{2}}.$$

**Proof.**  See Appendix E. □

Corollary 2 shows that the problem of finding a polynomial fit for f(s)=−slns on (0,b] is equivalent to finding the inverse of $\tilde{M}$, which is independent of *b*. However, $\tilde{M}$ is an ill-conditioned matrix as the elements ${\tilde{M}}_{ij}$ become increasingly smaller for higher i+j. For example, if we take r=−2, then $\tilde{M}$ is the Hilbert matrix with inverse elements, (H−1)ij=(−1)i+j(i+j−1)C+i−1C−jC+j−1C−ii+j−2i−12, which become numerically unstable at C∼10. If we write $f_{\max}\equiv \max_{x\in R^{n}}f_{X}\left(x\right)$, then for our Gaussian mixture $f_{X}$, the possible values are in $\left(0,f_{\max}\right]$. In a very large part of $R^{n}$, we find ourselves in the tails of the Gaussian mixture, which corresponds to $f_{X}\left(x\right)$ being close to zero. Hence, we want our weight function w(s) to reflect this, and emphasize the region close to zero in the application of Corollary 2. Note that we can write

(26) $$\begin{array}{cc} \; & h\left(X\right)=-\int f_{X}\left(x\right)\ln f_{X}\left(x\right)dx=-\int \left(\int_{0}^{f_{\max}}\delta \left(s-f_{X}\left(x\right)\right)s\ln s\;ds\right)dx\\ \; & =-\int_{0}^{f_{\max}}s\ln s\left(\int \delta (s-f_{X}\left(x\right))dx\right)ds=\int_{0}^{f_{\max}}f\left(s\right)V\left(s\right)\;ds, \end{array}$$

where $V\left(s\right)=\int \delta (s-f_{X}\left(x\right))dx$ can be thought of as the (n−1)-dimensional volume where the function $f_{X}$ equals *s*. We see from Equation (26) that our entropy approximation is not only determined by how well we can estimate f(s) using Corollary 2, but also by V(s), which for Gaussian mixtures will put more weight around s=0. That is, we expect r<0 in Corollary 2 to produce better results for our entropy approximation than r>0, although they will produce worse estimates of f(s) over any interval. Ideally, we set w(s)=V(s) in Lemma 3, but V(s) has no closed-form solution. This leads us to the following entropy approximation given by Theorem 2.

**Theorem 2.** 
*Let r>−3, $I=(0,f_{\max}]$; then, the entropy approximation ${\bar{h}}_{C}^{\mathrm{Polyfit}}\left(X\right)$ based on Lemma 3, Corollary 2, and Corollary 1 is given by the following:*

(27) h¯CPolyfit(X)=∑a=1CcaPolyfit∑t^∈Taat^∏i=1qpitiD(t^),

*where ${\vec{c}}^{\;\mathrm{Polyfit}}$ is given by the following:*

(28) $$c_{a}^{\mathrm{Polyfit}}=\left\{\begin{array}{cc} {\tilde{d}}_{1}-\ln f_{\max}, & \mathrm{if}\;a=1\\ f_{\max}^{1-a}{\tilde{d}}_{a}, & \mathrm{otherwise} \end{array}\right.,\;\vec{\tilde{d}}={\tilde{M}}^{-1}\vec{\tilde{z}},$$

*with ${\tilde{M}}_{ij}$ and ${\tilde{z}}_{i}$ given by the following:*

(29) $${\tilde{M}}_{ij}=\frac{1}{i+j+r+1},\;{\tilde{z}}_{i}=\frac{1}{{\left(i+r+2\right)}^{2}}.$$

**Proof.**  It follows from the direct application of Lemma 3, Corollary 2, and Corollary 1 to $-f_{X}\ln f_{X}$. □

In Appendix F, we derive the volume function V(s) for the trivial case q=1. Around s≈0, it blows up as 1/s, with some additional logarithmic divergence; this gives some intuition as to why we get good results at r≤−1.

## 3. Numerical Results

### 3.1. Polyfit Results

We now present results of Theorem 2 applied to different Gaussian mixtures in order to estimate h(X). The performance metric we use is the percentage error compared to the exact value for the entropy. The mixtures we used to show the validity of our approximation are shown in Table 1. The examples cover various values of *n* and *q*. This is not meant to be all-encompassing, but it still covers a sufficient part of the parameter space to showcase the accuracy of our approximation.

**Figure 1:**
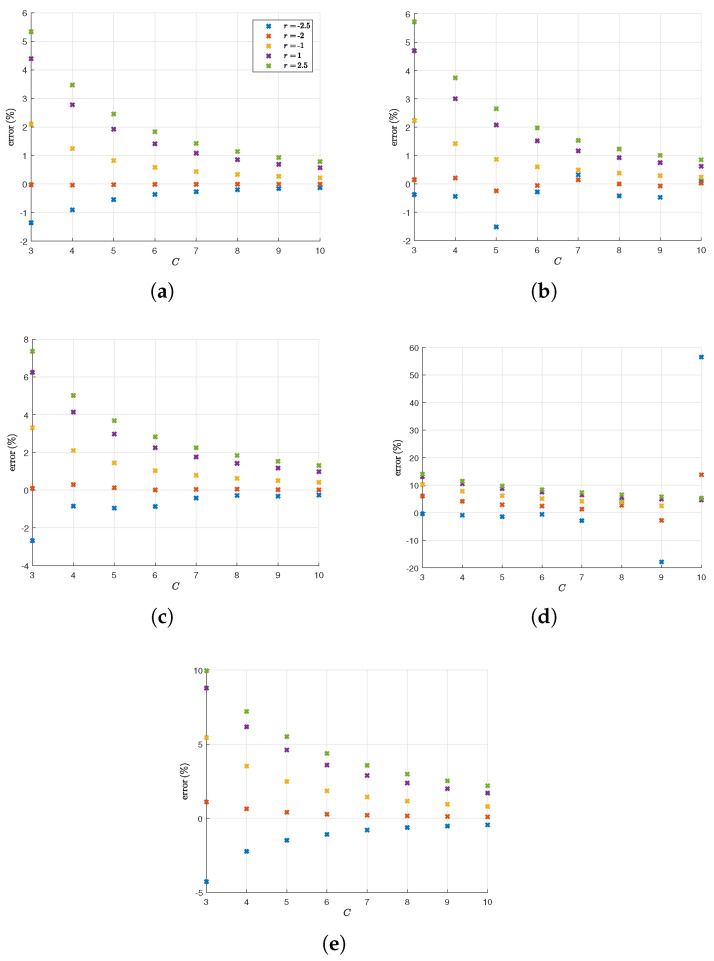
The results of testing Theorem 2 on the Gaussian mixtures in Table 1. The graphs show the percentage error $\left(\frac{h\left(X\right)-{\bar{h}}_{C}^{\mathrm{Polyfit}}\left(X\right)}{h(X)}\;100\right)$ of the approximation ${\bar{h}}_{C}^{\mathrm{Polyfit}}\left(X\right)$ at different values of *C*. (**a**) $q=3,n=2,K_{i}=I_{2}$; (**b**) q=3,n=2; (**c**) q=4,n=3; (**d**) q=4,n=8; (**e**) q=5,n=4.

From Figure 1, we see that for low-dimensional cases (q=3,n=2), our approximation converges quickly to the exact value of the entropy (within less than 1% error), and we can fairly trust numerical accuracy for those cases. As for the optimal *r* value for the weight function, r=−2 is consistently the best choice for those parameters, with r>0 being less favorable, as predicted in Section 2.3.2.

At q=4, we start to see divergent behavior as *n* becomes larger. For the lower-dimensional case of q=4,n=3, the behavior is similar to previous cases with very high accuracy and r=−2 being the most favorable weight function. However, in the case of q=4,n=8, the result diverges as *C* grows larger, although up to C=7, the approximation is fairly accurate. Furthermore, the best-performing *r* in this case is r=−2.5 with an error percentage of around −0.5% at C=6. We conjecture that the divergent behavior in this case is caused by numerical inaccuracies in the evaluation of multinomial coefficients.

For q=5,n=4, we again have convergent behavior yielding an accurate approximation of the entropy with r=−2 being the best weight function. This prompts us to conclude that our method produces a very highly accurate approximation when r∈[−2.5,−2] and *C* is kept between 3 and 8 in order to avoid computational inaccuracies. Note that the upper bound mentioned in the introduction $h\left(X\right)\leq \sum_{j=1}^{q}p_{j}\ln\frac{1}{p_{j}}+\sum_{j=1}^{q}p_{j}\frac{1}{2}\ln\det\left(2\pi eK_{j}\right)$ produces values that are within 3–19% above the true entropy for the configurations in Table 1.

**Figure 2:**
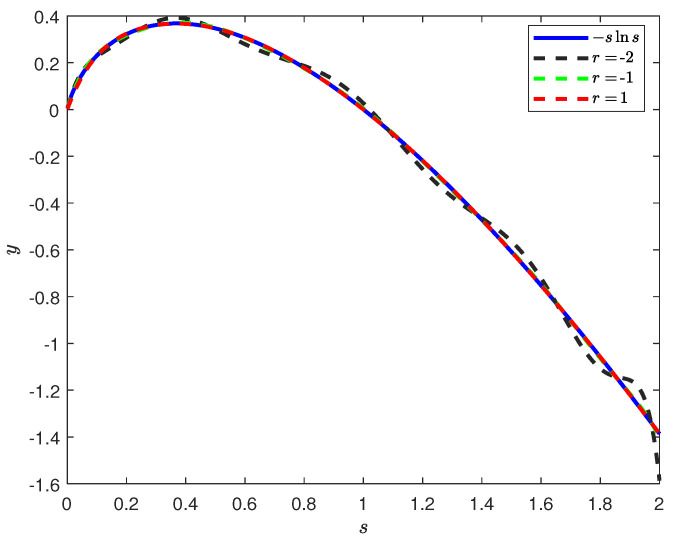
Polyfit approximations of f(s)=−slns for different values of *r* on the interval (0, 2] in accordance with Lemma 3.

In Figure 2, we show Polyfit approximations of f(s)=−slns as described in Lemma 3 for different values of *r*. We see clearly in Figure 2 that for r=−2, the approximation is overall inaccurate compared to r=−1 and r=1. However, r=−2 yields the best approximation for the entropy h(X). As we discussed in Section 2.3.2, the reason for this is that the value of r=−2 makes the approximation better around s≈0, which is where V(s) is highest.

### 3.2. Taylor Series Results

**Figure 3:**
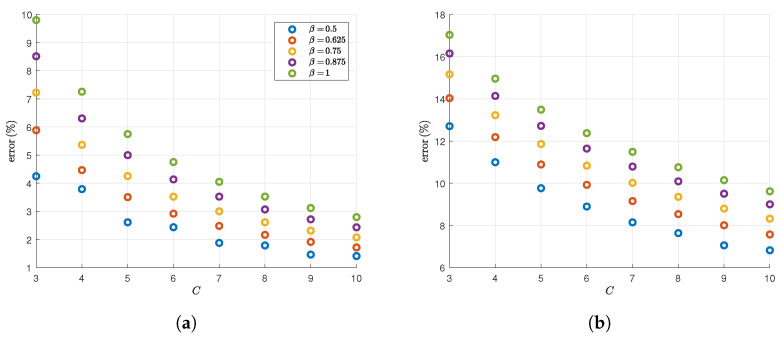
The results of testing Theorem 1 on two of the Gaussian mixtures in Table 1. The graphs show the percentage error $\left(\frac{h\left(X\right)-{\bar{h}}_{C,m}^{\mathrm{Taylor}}\left(X\right)}{h(X)}\;100\right)$ of the approximation ${\bar{h}}_{C,m}^{\mathrm{Taylor}}\left(X\right)$ at different values of *C* and for different values of β. (**a**) q=3,n=2; (**b**) q=4,n=8.

We now show results for our approximation ${\bar{h}}_{C,m}^{\mathrm{Taylor}}\left(X\right)$ based on the Taylor expansion of the logarithm as outlined in Theorem 1. We do not expect it to be as accurate as ${\bar{h}}_{C,m}^{\mathrm{Polyfit}}\left(X\right)$; however, it provides us with an easy-to-compute lower bound for the entropy, as mentioned earlier in Section 2.2. We show the percentage error compared to the exact value of the entropy for different values of β for two mixtures that we take from Table 1. We use the case of q=3,n=2 with non-spherical covariance matrices, as well as the case of q=4,n=8. The results are shown in Figure 3. As we can see from Figure 3, ${\bar{h}}_{C,m}^{\mathrm{Taylor}}\left(X\right)$ is not as accurate as ${\bar{h}}_{C,m}^{\mathrm{Polyfit}}\left(X\right)$ as expected, especially in the higher-dimensional case. However, it does provide monotonous and convergent behavior. Furthermore, the case β=1/2 is the fastest-converging case, which is also the case where computational inaccuracies start to appear the fastest as we increase *C*.

## 4. Discussion

Gaussian mixtures are one of the most applicable distributions in the literature, from wireless communications to physics and diffusion models. They have found success in either describing or modeling real life phenomena to a high degree of accuracy. One of the most important quantities in characterizing a distribution is the Shannon (differential) entropy, which, for Gaussian mixtures, does not have a known closed-form expression.

In this paper, we have derived a method by which the differential entropy of Gaussian mixtures can be approximated both accurately and efficiently. Our method enjoys a high degree of simplicity as it relies on the fact that for Gaussian mixtures “*almost all the support has an image that is almost zero*”. For various configurations, this trick allows our approximation to get very close to the true value for the differential entropy by summing only a small number of terms. Although some configurations are more difficult to approximate with a small number of terms, our method is accurate for a large number of randomly generated Gaussian mixtures (not reported in Section 3). This makes inaccuracy an exception that can be circumvented by avoiding numerical instabilities and taking into account higher-order terms.

It is not straightforward to benchmark against the existing literature. Many methods are at least partly numerical, whereas our approximation technique yields fully analytic expressions. The work [21] is closest to our technique in that it approximates h(X) with closed-form analytic expressions. However, there are significant qualitative differences. Since [21] tries to polynomially approximate lnf(x), the order *C* of their Taylor series has to increase with *q* as C=2q (in the worst case) in order to correctly fit all the ‘bumps’ in the function. The error in lnf(x) then explodes as ${\left|x\right|}^{2q}$ when |x| increases beyond a certain radius. This large error should be damped by the fact that it gets multiplied by the Gaussian tail of f(x). However, at large *q*, this can require drastic application of the splitting trick, which introduces errors. In contrast, our choice of polynomial order *C* is independent of *q*, and increasing *q* does not lead to errors. In our technique, inaccuracies are caused by the mismatch between the function $s\ln\frac{1}{s}$ and the polynomial fit around s=0. The performance of our technique is constrained by the fact that the function $s\ln\frac{1}{s}$ has infinite derivative at s=0 and hence cannot be fitted with a polynomial of finite degree at that point.

Our Polyfit method relies on choosing an appropriate weight function w(s) as discussed in Section 2.3. Ideally for Gaussian mixtures, w(s) should be optimally chosen as V(s), which is difficult to estimate. However, from our experiments, we notice that a simple power $w\left(s\right)\propto s^{r}$ gives good results, especially for negative *r* around r=−2. At negative *r*, a lot of weight is given to small values of *s*. This makes sense because most of the *n*-dimensional volume in the integral ∫dx covers the tails of the distribution f(X). For a single Gaussian with spherical symmetry, it is easy to verify (see Appendix F) that the volume function V(s)=∫dxδ(s−f(x)) behaves as $V\left(s\right)\propto \frac{1}{s}{\left(\ln\frac{f_{\max}}{s}\right)}^{\frac{n}{2}-1}$. At small *s*, this expression blows up faster than $\frac{1}{s}$ but slower than $\frac{1}{s^{2}}$. This suggests that *r* should lie in the vicinity of [−2,−1]. More extensive testing, with larger mixtures in higher dimensions, will show how generally applicable our method is. We have not exhaustively studied all possible polynomial fits. It is quite likely that some improvement can be gained by choosing a better weight function, e.g., one that more resembles V(s).

Projection pursuit was introduced in 1974 as a method for analyzing multivariate data through its lower-dimensional projections [23]. However, exploring the full space of projections becomes increasingly impractical as the dimension grows [24]. Subsequent work addressed this issue by identifying “interesting” projections, namely those that reveal structure in the original data, through indices that measure departures from Gaussianity. One such index is differential entropy [25]. When the data arise from a finite mixture, skewness has also been used as a measure of non-normality for classifying projections [26,27]. Other approaches include the differential entropy estimator proposed in [28]. It would be of interest to compare these approaches with accurate estimation of differential entropy, using our method and taking our estimate as the projection index. In particular, an important question is whether maximizing skewness also leads to entropy optimization. A similar investigation could be carried out in relation to the work of Peña and Prieto [29,30,31]. These directions are left for future work.

## Figures and Tables

**Table 1:** The different Gaussian mixtures tested using Theorem 2. The resulting entropy errors appear in Figure 1.

*q*	*n*	Mixture Parameters
3	2	$w_{1}={\left(1,0\right)}^{T},\;w_{2}={\left(-1,0\right)}^{T},\;w_{3}={\left(0,1.5\right)}^{T},\;K_{i}=I_{2},\;\hat{p}={\left(0.2,0.3,0.5\right)}^{T}$
	2	$w_{1}={\left(0,0\right)}^{T},\;w_{2}={\left(-1.5,1.5\right)}^{T},\;w_{3}={\left(1.5,1.5\right)}^{T},\;K_{1}=\left(\begin{array}{cc} 1 & 0\\ 0 & 3 \end{array}\right),\;K_{2}=\left(\begin{array}{cc} 1 & 0.2\\ 0.2 & 1 \end{array}\right)$, K3 = $\left(\begin{array}{cc} 1 & -0.2\\ -0.2 & 1 \end{array}\right)$, $\hat{p}={\left(0.2,0.3,0.5\right)}^{T}$
4	3	$w_{1}={\left(0,0,0\right)}^{T},\;w_{2}={\left(-1.5,1.5,-1.5\right)}^{T},\;w_{3}={\left(1.5,1.5,1.5\right)}^{T},\;w_{4}={\left(1,1,1\right)}^{T},\;K_{i}=I_{3},$ $\hat{p}={\left(0.2,0.3,0.3,0.2\right)}^{T}$
	8	$w_{1}={\left(0,\ldots ,0\right)}^{T},\;w_{2}={\left(-1.5,1.5,\ldots ,1.5\right)}^{T},\;w_{3}={\left(1.5,\ldots ,1.5\right)}^{T},\;w_{4}={\left(1,\ldots ,1\right)}^{T},\;K_{i}=I_{8},$ $\hat{p}={\left(0.2,0.3,0.3,0.2\right)}^{T}$
5	4	$w_{1}={\left(0,0,0,0\right)}^{T},\;w_{2}={\left(-1.5,1.5,-1.5,1.5\right)}^{T},\;w_{3}={\left(1.5,1.5,1.5,1.5\right)}^{T},\;w_{4}={\left(1,1,1,1\right)}^{T}$
		$w_{5}={\left(-3,3,-3,3\right)}^{T},\;K_{i}=I_{4},\;\hat{p}={\left(0.2,0.3,0.3,0.1,0.1\right)}^{T}$

## Data Availability

The original contributions presented in this study are included in the article. Further inquiries can be directed to the corresponding author.

## Appendix A. Proof of Lemma 1

(A1) $$\begin{array}{cc} \; & \int \prod_{j=1}^{q}g_{j}^{t_{j}}\left(x\right)dx=\int \prod_{j=1}^{q}{\left({\left(2\pi \right)}^{-\frac{n}{2}}{\left(\det K_{j}\right)}^{-1/2}e^{-\frac{1}{2}{\left(x-w_{j}\right)}^{T}K_{j}^{-1}\left(x-w_{j}\right)}\right)}^{t_{j}}dx\\ \; & =C\left(\hat{t}\right)\int e^{-\frac{1}{2}\sum_{j=1}^{q}t_{j}{\left(x-w_{j}\right)}^{T}K_{j}^{-1}\left(x-w_{j}\right)}dx\\ \; & =C\left(\hat{t}\right)e^{-\frac{1}{2}\sum_{j=1}^{q}t_{j}w_{j}^{T}K_{j}^{-1}w_{j}}\int e^{-\frac{1}{2}\sum_{j=1}^{q}t_{j}\left(x^{T}K_{j}^{-1}x-x^{T}K_{j}^{-1}w_{j}-w_{j}^{T}K_{j}^{-1}x\right)}dx\\ \; & =C\left(\hat{t}\right)e^{-\frac{1}{2}\sum_{j=1}^{q}t_{j}w_{j}^{T}K_{j}^{-1}w_{j}}\int e^{-\frac{1}{2}\left(x^{T}\left(\sum_{j=1}^{q}t_{j}K_{j}^{-1}\right)x-x^{T}\left(\sum_{j=1}^{q}t_{j}K_{j}^{-1}w_{j}\right)-\left(w_{j}^{T}\sum_{j=1}^{q}t_{j}K_{j}^{-1}\right)x\right)}dx\\ \; & =C\left(\hat{t}\right)e^{-\frac{1}{2}\sum_{j=1}^{q}t_{j}w_{j}^{T}K_{j}^{-1}w_{j}}\int e^{-\frac{1}{2}\left(x^{T}M^{-1}x-x^{T}M^{-1}\mu -\mu^{T}M^{-1}x\right)}dx\\ \; & =C\left(\hat{t}\right)e^{-\frac{1}{2}\sum_{j=1}^{q}t_{j}w_{j}^{T}K_{j}^{-1}w_{j}}e^{\frac{1}{2}\mu^{T}M^{-1}\mu }\int e^{-\frac{1}{2}{\left(x-\mu \right)}^{T}M^{-1}\left(x-\mu \right)}dx\\ \; & =C\left(\hat{t}\right)e^{-\frac{1}{2}\sum_{j=1}^{q}t_{j}w_{j}^{T}K_{j}^{-1}w_{j}}e^{\frac{1}{2}\mu^{T}M^{-1}\mu }{\left(2\pi \right)}^{n/2}{\left(\det M\right)}^{1/2}, \end{array}$$

where we have the following:

(A2) $$\begin{array}{c} C\left(\hat{t}\right)=\prod_{j=1}^{q}{\left(2\pi \right)}^{-nt_{j}/2}{\left(\det K_{j}\right)}^{-t_{j}/2}={\left(2\pi \right)}^{-na/2}\prod_{j=1}^{q}{\left(\det K_{j}\right)}^{-t_{j}/2}. \end{array}$$

Note that we can alternatively write:

(A3) $$\mu^{T}M^{-1}\mu ={\left(\sum_{j=1}^{q}t_{j}K_{j}^{-1}w_{j}\right)}^{T}M\left(\sum_{l=1}^{q}t_{l}K_{l}^{-1}w_{l}\right).$$

## Appendix B. Proof of Corollary 1

(A4) ∫fXa(x)dx=∫∑i=1qpigi(x)adx=∫∑t1+t2+⋯+tq=at1,t2,…,tq≥0at1,t2,…,tq∏i=1qpiti∏j=1qgjtj(x)dx=∑t1+t2+⋯+tq=at1,t2,…,tq≥0at1,t2,…,tq∏i=1qpiti∫∏j=1qgjtj(x)dx=∑t1+t2+⋯+tq=at1,t2,…,tq≥0at1,t2,…,tq∏i=1qpitiD(t^),

where the last equality follows from Lemma 1. Plugging into Equation (4) gives the desired expression.

## Appendix C. Proof of Theorem 1

(A5) fXZk=fX∑b=0kkb−m−1fXb=∑b=0kkb(−m)−bfXb+1=−m∑b=0kkb(−1)b+1fXb+1mb+1=−m∑a=1k+1ka−1(−1)afXama,

and plugging into Equation (10), we have the following:

(A6) −fXlnfXm=∑k=1∞−mk∑a=1k+1ka−1(−1)afXama=∑k=1∞∑a=1k+1c˜k,afXa,

where ${\tilde{c}}_{k,a}$ is given by the following:

(A7) c˜k,a=ka−1(−1)a+1kma−1.

If we now take the first C−1 terms, we have the following:

(A8) $$\begin{array}{cc} \; & -f_{X}\ln\frac{f_{X}}{m}=\sum_{k=1}^{C-1}\sum_{a=1}^{k+1}{\tilde{c}}_{k,a}f_{X}^{a}+\cdots =\sum_{k=1}^{C-1}\sum_{a=1}^{C}{\tilde{c}}_{k,a}f_{X}^{a}+\cdots =\sum_{a=1}^{C}\sum_{k=1}^{C-1}{\tilde{c}}_{k,a}f_{X}^{a}+\cdots \\ \; & =\sum_{a=1}^{C}c_{a}^{\mathrm{Taylor}}f_{X}^{a}+\cdots \end{array}$$

where we have the following:

(A9) $$c_{1}^{\mathrm{Taylor}}=\sum_{k=1}^{C-1}{\tilde{c}}_{k,1}=H_{C-1},$$

where $H_{k}$ is the *k*-th Harmonic number. Furthermore, we have (a≠1):

(A10) caTaylor=∑k=1C−1c˜k,a=∑k=1C−1ka−1(−1)a+1kma−1=∑k=a−1C−1ka−1(−1)a+1kma−1=(−1)a+1ma−11a−1C−1a−1,

and the last equality follows from the following:

(A11) ∑k=aC1kka=∑k˜=0C−a1k˜+ak˜+aa=∑k˜=0C−a1ak˜+a−1a−1=1aCa,

where we used the binomial identities:

(A12) 1aab=1ba−1b−1,a,b≠0,

(A13) ∑j=0mn+jn=n+m+1n+1=n+m+1m.

Plugging the coefficients $\left\{c_{a}^{\mathrm{Taylor}}\right\}$ into Corollary 1, we have the following:

(A14) h(X)≈−lnm+HC−1∑t1+t2+⋯+tq=1t1,t2,…,tq≥01t1,t2,…,tq∏i=1qpitiD(t^)+∑a=2C(−1)a+1ma−11a−1C−1a−1∑t1+t2+⋯+tq=at1,t2,…,tq≥0at1,t2,…,tq∏i=1qpitiD(t^)=−lnm+HC−1+∑a=1C−1(−1)ama1aC−1a∑t1+t2+⋯+tq=a+1t1,t2,…,tq≥0a+1t1,t2,…,tq∏i=1qpitiD(t^)=−lnm+HC−1−m∑a=1C−1Ba+1aC−1a,

where we used:

(A15) ∑t1+t2+⋯+tq=1t1,t2,…,tq≥01t1,t2,…,tq∏i=1qpitiD(t^)=1.

## Appendix D. Proof of Lemma 2

If we take the partial derivative of *E* w.r.t $d_{I,i}$, we get the following:

(A16) $$\begin{array}{cc} \; & \frac{\partial E}{\partial d_{I,i}}=\frac{-2}{b-a}\int_{a}^{b}w\left(s\right)\left(f\left(s\right)-\hat{f}\left(s\right)\right)s^{i}ds\\ \; & =\frac{-2}{b-a}\left(\int_{a}^{b}w\left(s\right)f\left(s\right)s^{i}ds-\sum_{j=1}^{C}d_{I,j}\int_{a}^{b}w\left(s\right)s^{i+j}ds\right), \end{array}$$

and setting it to zero, we get the following:

(A17) $$\sum_{j=1}^{C}d_{I,j}\int_{a}^{b}w\left(s\right)s^{i+j}ds=\int_{a}^{b}w\left(s\right)f\left(s\right)s^{i}ds,$$

or written in matrix form as follows:

(A18) $$M_{I}{\vec{d}}_{I}={\vec{z}}_{I}.$$

In order to show that our solution is a global minimum, we show that the Hessian matrix $H_{ij}=\frac{\partial E}{\partial d_{I,i}\partial d_{I,j}}$ is positive definite. Taking the partial derivatives, we have the following:

(A19) $$H_{ij}=\frac{2}{b-a}\int_{a}^{b}w\left(s\right)s^{i}s^{j}ds,$$

which is a Gram matrix (note that w(s)>0 and a Gram matrix is positive semi-definite). It is straightforward to see that *H* is positive definite since ${\left\{\sqrt{w(s)}s^{i}\right\}}_{i=1}^{C}$ are linearly independent.

## Appendix E. Proof of Corollary 2

From Lemma 3 and Equation (20), we have the following:

(A20) $$\begin{array}{cc} \; & \sum_{j}{\left(M_{I}\right)}_{ij}{\left({\vec{d}}_{I}\right)}_{j}={\left({\vec{z}}_{I}\right)}_{i}\Rightarrow \sum_{j}\frac{b^{i+j+r+1}}{i+j+r+1}{\left({\vec{d}}_{I}\right)}_{j}=\frac{b^{i+r+2}}{{\left(i+r+2\right)}^{2}}-\frac{b^{i+r+2}\ln b}{i+r+2}\\ \; & \Rightarrow \sum_{j}\frac{b^{i+j+r+1}}{i+j+r+1}{\left({\vec{d}}_{I}\right)}_{j}+\frac{b^{i+r+2}\ln b}{i+r+2}=\frac{b^{i+r+2}}{{\left(i+r+2\right)}^{2}}\\ \; & \Rightarrow \sum_{j\neq 1}\frac{b^{i+j+r+1}}{i+j+r+1}{\left({\vec{d}}_{I}\right)}_{j}+\frac{b^{i+r+2}}{i+r+2}{\left({\vec{d}}_{I}\right)}_{1}+\frac{b^{i+r+2}\ln b}{i+r+2}=\frac{b^{i+r+2}}{{\left(i+r+2\right)}^{2}}\\ \; & \Rightarrow \sum_{j\neq 1}\frac{b^{j-1}}{i+j+r+1}{\left({\vec{d}}_{I}\right)}_{j}+\frac{{\left({\vec{d}}_{I}\right)}_{1}+\ln b}{i+r+2}=\frac{1}{{\left(i+r+2\right)}^{2}}\\ \; & \Rightarrow \sum_{j\neq 1}\frac{b^{j-1}}{i+j+r+1}{\left({\vec{d}}_{I}\right)}_{j}+{\left.\left(\frac{b^{j-1}{\left({\vec{d}}_{I}\right)}_{j}+\ln b}{i+j+r+1}\right)\right|}_{j=1}=\frac{1}{{\left(i+r+2\right)}^{2}}\\ \; & \Rightarrow \sum_{j}{\tilde{M}}_{ij}{\tilde{d}}_{j}={\tilde{z}}_{i}. \end{array}$$

## Appendix F. Volume *V*(*s*) for a Single Gaussian

We consider the trivial ‘mixture’ that consists of a single Gaussian centered on the origin, with spherical covariance matrix $\sigma^{2}1$. The volume V(s) as defined in (26) is then given by

(A21) $$V\left(s\right)=\int dx\;\delta \left(s-N_{0,\sigma^{2}1}\left(x\right)\right)\propto \int_{0}^{\infty }dr\;r^{n-1}\delta \left(s-\frac{\exp-\frac{r^{2}}{2\sigma^{2}}}{{\left(\sigma \sqrt{2\pi }\right)}^{n}}\right).$$

We apply the rule $\delta \left(s-y\left(r\right)\right)=\frac{\delta (r-y^{\mathrm{inv}}\left(s\right))}{|y^{\prime }\left(r\right)|}$ and obtain

(A22) $$\begin{array}{cc} \; & V\left(s\right)\propto \int_{0}^{\infty }dr\;r^{n-1}\frac{\delta \left(r-\sqrt{-2\sigma^{2}\ln\left[s{\left(\sigma \sqrt{2\pi }\right)}^{n}\right]}\;\right)}{\frac{\exp-\frac{r^{2}}{2\sigma^{2}}}{{\left(\sigma \sqrt{2\pi }\right)}^{n}}\cdot \frac{r}{\sigma^{2}}}\propto \frac{1}{s}{\left(\sqrt{\ln\frac{1}{s{\left(\sigma \sqrt{2\pi }\right)}^{n}}}\right)}^{n-2}\\ \; & =\frac{1}{s}{\left(\ln\frac{f_{\max}}{s}\right)}^{\frac{n}{2}-1}. \end{array}$$

Here, we have used that $\frac{\exp-\frac{r^{2}}{2\sigma^{2}}}{{\left(\sigma \sqrt{2\pi }\right)}^{n}}=s$ and that $f_{\max}=\frac{1}{{\left(\sigma \sqrt{2\pi }\right)}^{n}}$.
